# Fast, Multi-Dimensional and Simultaneous Kymograph-Like Particle Dynamics (SkyPad) Analysis

**DOI:** 10.1371/journal.pone.0089073

**Published:** 2014-02-19

**Authors:** Bruno Cadot, Vincent Gache, Edgar R. Gomes

**Affiliations:** 1 UMRS 787 INSERM, Université Pierre et Marie Curie Paris 6, Paris, France; 2 Groupe Hospitalier Pitié-Salpêtrière, Institut de Myologie, Paris, France; University of Toronto, Canada

## Abstract

**Background:**

Kymograph analysis is a method widely used by researchers to analyze particle dynamics in one dimensional (1D) trajectories.

**Results:**

Here we provide a Visual Basic-coded algorithm to use as a Microsoft Excel add-in that automatically analyzes particles in 2D trajectories with all the advantages of kymograph analysis.

**Conclusions:**

This add-in, which we named SkyPad, leads to significant time saving and higher accuracy of particle analysis. Finally, SkyPad can also be used for 3D trajectories analysis.

## Background

**Figure 1:**
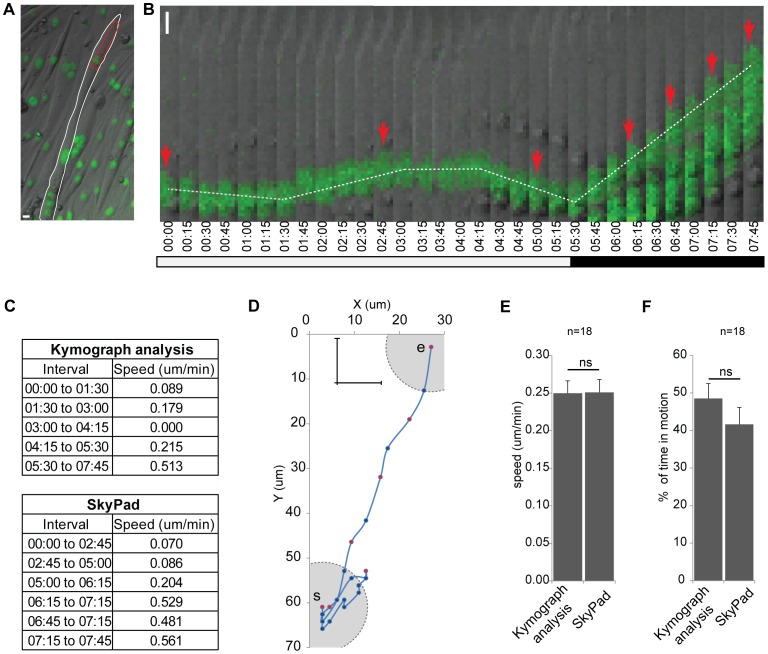
Comparison between kymograph analysis and SkyPad. **A**: First frame of a movie showing a myotube (white outline) with GFP-H1 nuclei (green). The red rectangle corresponds to the region used to create the kymograph in panel b. **B**: Kymograph showing nuclei moving in a myotube. Red arrows indicate the position where the nucleus moved at least 10 um from the previous arrow as calculated through SkyPad. White bar indicates a pausing or oscillating period, black bar indicates a moving period. Time in HH:min **C**: Speeds obtained by kymograph analysis and SkyPad between indicated frames. **D**: Trajectory of the nucleus represented with the coordinates in X and Y at each time point. The cross and gray circle represent the 10 um distance threshold used in SkyPad. The circle is traced on the first and last point of the trajectory (s and e respectively). Red dots correspond to arrow pointed positions in panel b. **E, F**: Speeds (e) and percentage of time in motion (f) of 18 nuclei were analyzed using kymograph and skypad analysis. Error bars correspond to S.E.M. Scale bars in a and b: 10 um.

The measurement of particle dynamics is a powerful tool to understand fundamental mechanisms in cell biology. Multiple automated methods, commercial and non-commercial, are available to track particles and measure particle dynamics [1], [2]. The averaging of instantaneous speeds between each position is usually used to study particle dynamics [3], [4]. However, errors arise when particles oscillate and no significant displacement occurs, although instantaneous speeds are still measured. In these situations, the instantaneous speeds of oscillating particles can be similar to the instantaneous speeds of moving particles. Furthermore, by averaging of instantaneous speeds, it is not possible to calculate the percentage of time in motion of particles. To overcome these errors, kymograph analysis is commonly used to extract speed values manually from segments where particles are moving linearly, i.e., in one dimension (1D) [5]–[7]. Kymographs are built by delimitating a rectangular region aligned along the axis of particle movement in each image of a time-lapse series (Figure 1a). The length and width of the rectangle are determined by the area occupied by the particle track. These regions in each image are then extracted and sequentially aligned side-by-side and lines are manually drew along the analyzed particles (Figure 1b) [7]. The average speed is measured from the slopes of lines. The percentage of time in motion of particles can also be calculated by a user-defined speed threshold. The inconvenient of this technique resides on the multiple manual steps that are time-consuming and the difficulty of using kymographs to analyze particles moving in two- and three-dimensions [2]. An algorithm, combining the advantages of the particle tracking methods with the advantages of the kymograph analysis would provide a faster and accurate method to retrieve particles dynamics measurements.

## Results and Discussion

### Algorithm description

We describe here an automated algorithm which analyzes particle coordinates obtained by any tracking method over time, with similar results to the manual kymograph analysis but with an execution time roughly 100 times faster. This algorithm, that we named SkyPad (for Simultaneous Kymograph-like Particle dynamics analysis), calculates distance and speed between particle positions using the particle coordinates. SkyPad calculates the distance between positions at frames n and n+x until the distance between n and n+x is higher than a user-defined distance threshold (DT). DT corresponds to a displacement considered as significant by the user, such as the size of the analyzed particles, and is similar to the threshold used by users in kymograph analysis, when drawing the lines along the trajectory particle. Then, once this criterion is met, the speed between n and n+x is calculated. If the speed is below a user-defined speed threshold (ST), the particle is considered as paused. If the speed is above the ST, then the particle is considered as moving. The ST is similar to the speed threshold defined by users in kymograph analysis. The algorithm then restarts the calculation from the last frame (n+x). These values are then used to calculate the average speed when a particle is in motion, the percentage of time in motion and the number and duration of pauses, as measured in kymograph analysis. In addition, SkyPad also calculates the persistence of movement, the mean square displacement curve and a simple diffusion coefficient [8]. Persistence is defined as the ratio between the distance between the first and last position of a particle and the actual displacement of a particle. This definition of persistence is the same as chemotactic index and D/T directionality ratio commonly used to quantify polarized cell migration [9], [10]. Persistence in this context will illustrate the movement sustainability in one direction [10].

SkyPad is provided as a user friendly add-in for Excel spreadsheets (https://sites.google.com/site/cadotbruno/algorithms) and can be used in any Excel spreadsheet containing xy or xyz coordinates from particle tracking of any length. Furthermore, SkyPad is editable, easy access and can be used in a large range of applications among the scientific community, therefore fulfilling the guidelines recently proposed for the use of bioimaging software [11].

### Skypad versus Kymograph analysis

To exemplify SkyPad, we used kymograph and SkyPad analysis to measure nuclear movement in a multinucleated myotube (Figure 1a). In this example the nucleus oscillates during the initial 5 hour recording. Then, the nucleus moves unidirectionally until the end of the recording at 7 h45 min (Figure 1b). We calculated the nuclear movement speed using kymograph analysis by measuring the slopes of the white dashed lines (Figure 1b, c). We also determined the X and Y coordinates for each frame of the time-lapse movie (Figure 1d) and used SkyPad to analyze the movement, with a DT of 10 um (approximately the size of a nucleus), and a ST of 0.20 um/min (maximum speed measured by kymograph analysis and Skypad during oscillation, i.e. during the initial 5 h recording). SkyPad identified 6 segments where the nucleus was displaced above the DT (delimited by arrows in Figure 1b, 1c, red dots in Figure 1d). In the first 5 hours both analysis showed speed values below 0.20 um/min while after, kymograph and SkyPad analysis measured the same mean speed of 0.51 um/min. We performed the same analysis in an additional 18 nuclei and calculated the mean speeds and percentages of time in motion obtained using kymographs and SkyPad (Figure 1e, f, Figure S1). No differences were found between kymograph and SkyPad analysis (mean speed, p = 0.97; time in motion, p = 0.2, Student t-test), although SkyPad analysis was faster to perform and less prone to user errors. In addition SkyPad also automatically provided other parameters of particles dynamics such as the number and duration of pauses (similar to kymograph analysis), persistence, mean square displacement and diffusion coefficient (that cannot be calculated from kymograph analysis) [5], [12]–[14]. These parameters enhance the characterization and classification of particle dynamics (Figure S1b).

### Using Skypad to analyze 2D trajectories

**Figure 2:**
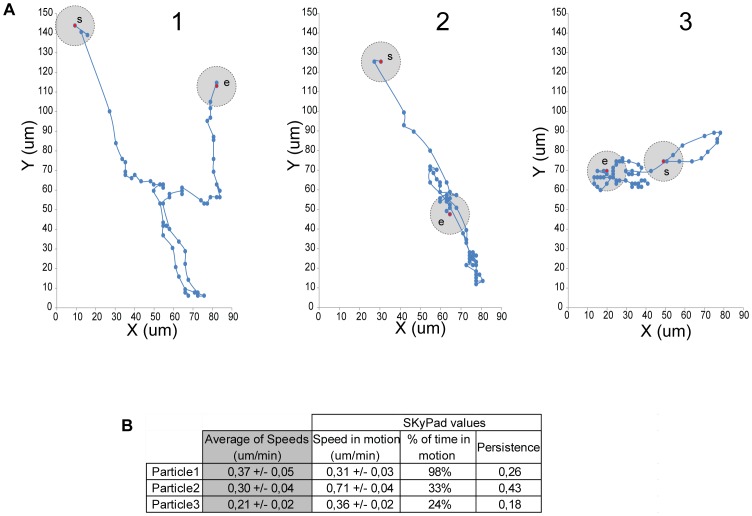
SkyPad analysis identifies multiple particles dynamics phenotypes. **A**: XY coordinates plots of 3 different particles. The gray circle on each plot represents the distance threshold used in the algorithm (10 um radius) and is showed on the first and last point of the trajectory. **B**: Table with the results obtained by averaging instantaneous speeds (gray column) and the results obtained by SkyPad (white columns) for the three particles described in a.

When a particle follows a two- or three-dimension trajectory, kymograph analysis is possible but becomes too laborious and prone to errors [2], [15]. Therefore averaging instantaneous speed is commonly used to analyze dynamics of two- or three-dimension trajectories. We used SkyPad to analyze the dynamics of particles moving in two dimensions and compared to instantaneous speed analysis. We choose 3 particles with different behaviors as illustrated by plotting their coordinates (Figure 2a, Movie S2). Particle 1 is always moving. Particle 2 moves faster than particle 1 but pauses for long periods. Particle 3 also pauses for long periods, but in the non-pausing periods, it moves as fast as particle 1. We analyzed the movement of these particles using the average of instantaneous speeds and SkyPad (Figure 2b). Particle 1 average instantaneous speeds is slightly higher than particle 2 but this analysis did not identify the long pauses and high speed of particle 2. On the other hand, SkyPad analysis identified that particle 1 was moving all the time (98% of time in motion) with a speed of 0,31 um/min, and particle 2 paused for longer periods (33% of time in motion) but moved faster (0,71 um/min) (Figure S2). Also, instantaneous speeds of particle 1 and 3 suggested a difference in the speed of particle movement (0,37 um/min vs 0,21 um/min, respectively). However, SkyPad analysis allow us to conclude that this difference is due to longer time in motion of particle 1 (98%) vs 24% of particle 3, while the speed both particles when they moved was similar (0,31 um/min vs 0,36 um/min, respectively) which suggests a different mechanism of movement (Figure S2). Therefore SkyPad can be used to analyze the dynamics of particle moving in 2D with similar parameters obtained by kymograph analysis (speed and time in motion) that leads to a better characterization of particle dynamics not possible to obtain by measuring instantaneous speeds.

SkyPad uses XY coordinates (2D trajectories) to analyze particle dynamics. The algorithm used by SkyPad can be used to analyze XYZ coordinates (3D trajectories), therefore we also provide an option within SkyPad to analyze XYZ coordinates, using the same principles and algorithm described above.

## Conclusions

Overall, we are providing a new tool to analyze particle dynamics, using particle coordinates obtained by automatic or manual tracking. SkyPad calculates the same parameters of kymograph analysis but with great advantage of automation and fast analysis of multiple particle tracks of any length, and applicability to 2D and 3D trajectories. The output parameters calculated by SkyPad allow a better characterization of particle dynamics and can lead to the identification of new mechanisms of particle displacement not identified by other particle dynamics analysis methods (Figure S3).

## Methods

### Cell culture and myotube formation

C2C12 cells obtained from ATCC (CRL-1772) were cultured in DMEM with 10% Fetal Bovine Serum in 5% CO2 and 37°C humidified incubator. Differentiation was induced by switching to DMEM with 2% horse serum when cells were at 70% confluence, as previously described [16].

### Skypad algorithm

SkyPad add-in is a free software: it can be redistributed and/or modified under the terms of the GNU General Public License as published by the Free Software Foundation, either version 3 of the License, or any later version.

SkyPad is distributed in the hope that it will be useful, but WITHOUT ANY WARRANTY; without even the implied warranty of MERCHANTABILITY or FITNESS FOR A PARTICULAR PURPOSE. See the GNU General Public License for more details.

A copy of the GNU General Public License (gpl.txt) should be distributed along with this macro. If not, see <http://www.gnu.org/licenses/>.

Distances in the algorithm are calculated as follow:
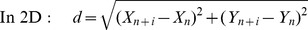


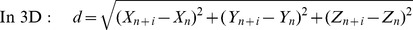
Where n is the starting frame and i the number of frames to increment until d reach the distance threshold DT. X, Y and Z correspond to the positions in the X, Y and Z axis.

Persistence is calculated as follow:
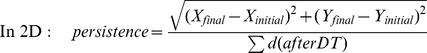


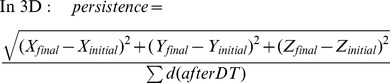
MSD was calculated as follow for each time interval (n*TF):
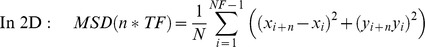


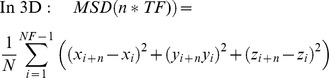
Where NF is the total number of frames, TF is the time frame, N the total number of increments measured for each time intervals.

The diffusion coefficient was calculated as follow:

First, for each time interval:
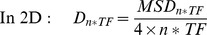


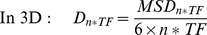
Then
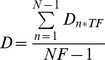



### Installation of Skypad Excel Add-In

From https://sites.google.com/site/cadotbruno/algorithms, download the version that suits for your operating system:

Windows version (SkyPad.xlam)
MacOS version (SkyPadMacOS.xla)

In Windows:

In Excel 2010:
  Go to Excel options, enable ’Show Developer tab in the Ribbon’ and click ‘OK’.
  Click on ‘Developer’ to show the developer ribbon. Select ‘complementary macros’. A new window appears, click on ‘browse’ and choose the folder where you download the macro, select it and then click ‘OK’. Close Excel

In Excel 2007 and previous versions:
  Go to ‘Excel options’, then to ‘add ins’ tab. On the bottom, be sure that ‘excel Add-ins’ is selected in ‘Manage’: and then click on ‘Go’. In the new window named ‘add-ins’, click on ‘browse…’ and choose the folder where you placed the downloaded macro, select it and then click ‘OK’. Click ‘OK’.

In MacOS

Excel 2011:
  Go to ‘Tools’ and select ‘Add-Ins’. Click on ‘select’ and choose the folder where you download the Add-In, select it (SkyPadMacOS.xla) and then click ‘OK’.

Excel 2004:
  Go to ‘Tools’ and select “complementary macros”. Choose the folder where you download the macro, select it and then click ‘OK’.

In some cases during installation, an error will appear concerning the libraries. Unselect all the libraries associated with the error (usually the last two in the list).
Also, an error “data may have been lost” can appear as Excel starts. It is un-harmful and you can just proceed.
Unfortunately, Excel 2008 in MacOS does not allow you to use complementary add-ins,

After installation, as soon as you open the Excel spreadsheet with the data to be analysed, you will have access to SkyPad by pressing **Ctr+SHIFT+S** in Windows or **Cmd+Alt+S** in MacOS.

### Formatting data to be used with SkyPad

You can use any method to track your particles. The information required for SkyPad must be organized in columns corresponding to X and Y coordinates and the frames numbers. In the provided example (test 18 nuclei.xls), frame numbers are in column A, X coordinates in column B and Y coordinates in C. Results from the same examples are shown in the sheet “expected results”.

Information in column D (particle) is not required and does not interfere with the analysis. It is use to better visualize the data corresponding to each particle to be analyzed.

The analysis starts at row 2 so leave the first row empty or with the description of your parameters.

Multiple particle tracks can be simultaneously analyzed by placing their coordinates and frame number sequentially in the same columns. The number of frames of each particle must be the same. In this example, each analyzed particle has 50 frames, therefore in column A, line 2 the first frame number is ‘1’. Sequential frame numbers must be inserted up to ‘50’, in line 51. The data regarding the second particle to be analyzed starts in line 52 and ends in line 101.

### Using SkyPad

Open the excel spreadsheet containing the tracking.

To run this macro just press Ctr+shift+S in Windows and Cmd+alt+S in MacOS and a new window will appear asking you for multiple parameters (Sup Figure 3A):

1. letter of the column where your X coordinates are.
2. letter of the column where your Y coordinates are.
3. letter of the column where your frames numbers are
4. length unit of your tracking data.
5. If you coordinates are in pixels, put here the ratio distance/pixel to convert pixels into the length unit you selected in “4”.
6. time unit of your tracking data
7. distance threshold in your length unit.
8. speed threshold in your length/time units.
9. number of frames of your tracking data
10. time between frames in the time unit selected in “6”
11. number of particles to analyze.
12. If you have 3D trajectories, check that box.
13. the column where your Z coordinates are.
14. Check that box if you want to see the plots of individual tracks before and after Skypad.
15. Check that box if you want to calculate the persistence

### Output excel file (Figure S3b)

The columns you chose as X and Y will be highlighted in gray, to allow easy verification of the selected columns.,

#### Columns R to X (Salmon color)

Intermediate calculations: distances, speed and coordinates. In red, you have the distance made by the particle over the first threshold. Next to them, you have the corresponding speed. In green, you have the speeds that are over the second threshold. If you want, you can go back in the several steps of the algorithm calculation to verify the process.

#### Columns Y to AE

Individual tracks if you choose to see them. The original trajectory and the positions isolated after SkyPad will be displayed.

#### Columns AF to AN

Analysis for each particle.

#### Black area

Parameters used for this analysis. These parameters will be automatically put in the SkyPad initial window for re-use.

#### Green area

Compilation of the analysis of all particles. If number of particles is below 15, standard deviation is showed. If number of particle is equal or more than 15, standard error of the mean is showed. This region will be automatically selected at the end of analysis to allow a fast copy and paste of the results to another spreadsheet. The average MSD of all particles for each time intervals and the corresponding chart is displayed below the green area.

#### Columns AQ and further

Calculations of the MSD for each particle (average in blue) and time intervals (in red) together with the diffusion coefficents (in gray).

Updates will be available on https://sites.google.com/site/cadotbruno/algorithms.

## Supporting Information

Figure S1 **A**: The kymographs analyzed to produce the 18 trajectories measured in Figure 1 e, f. Each segment analyzed is represented by a horizontal bar. The time frame is 20 minutes. **B**: Parameters retrieved by SkyPad characterizing the 18 nuclei.
(TIF)

Figure S2 **To compare particles behavior, the left panels represent the cumulative distance of raw position (blue line), the instantaneous speeds (red bars) and their average; the right panel shows the cumulative distance of positions corresponding to significative displacements (blue line), the speed for these displacements (red bars), the two types of periods (black and white bar and the values of speed and percentage of time in motion) obtained by Skypad analysis.**
(TIF)

Figure S3 **A:** After pressing Ctrl+Shit+S or Cmd+Alt+S this window will appear in Windows (left panel) or MacOS (right panel). **B:** After analysis, the excel spreadsheet will be updated with the results.
(TIF)

Movie S1 **Time-lapse movie used in** **figure 1** **of nuclei moving in myotubes.** The nucleus is indicated with a white arrow.
(AVI)

Movie S2 **Montage of time-lapse movies used in** **figure 2** **.** Each nucleus is indicated as colored dot. Trajectories are also indicated.
(AVI)

Table S1 We provide a test excel file with the 18 nuclei trajectories coordinates used in Figure 1: test 18 nuclei.xlsx. A protected spreadsheet is also included showing the expected results with parameters used in the article. SkyPadWindows.xlam is the complementary macro to install in Windows operating computers. SkyPadMacOS.xla is the complementary macro to install in Mac operating computers.
(XLS)
