# Supplementary figures and images for: Fast, Multi-Dimensional and Simultaneous Kymograph-Like Particle Dynamics (SkyPad) Analysis
Source: PLoS One. 2014 Feb 19;9(2):e89073. doi: 10.1371/journal.pone.0089073 (PMC3929634; doi:10.1371/journal.pone.0089073)

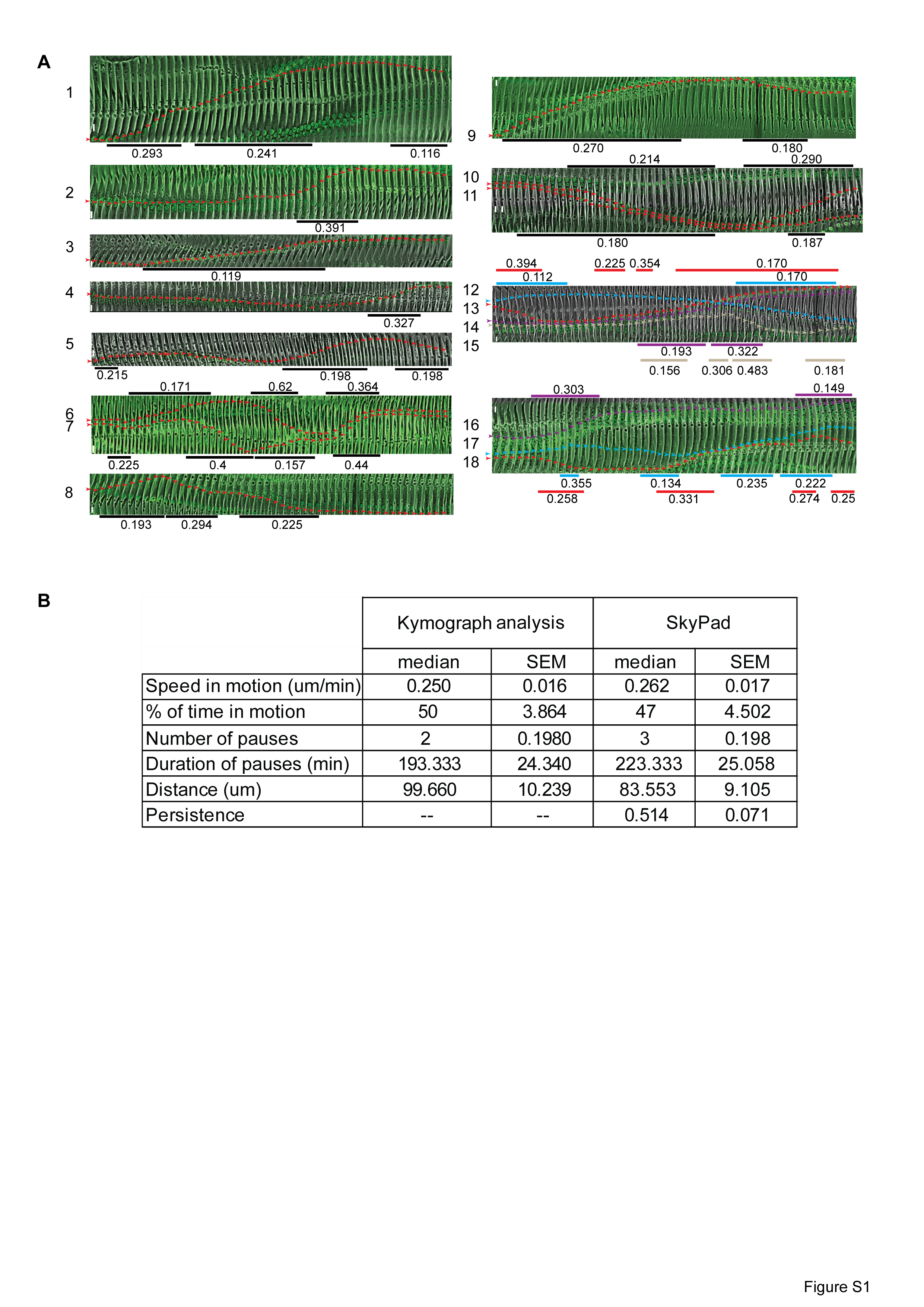

Supplement: Figure S1 — A: The kymographs analyzed to produce the 18 trajectories measured in Figure 1 e, f. Each segment analyzed is represented by a horizontal bar. The time frame is 20 minutes. B: Parameters retrieved by SkyPad characterizing the 18 nuclei. (TIF) [file pone.0089073.s001.tif]

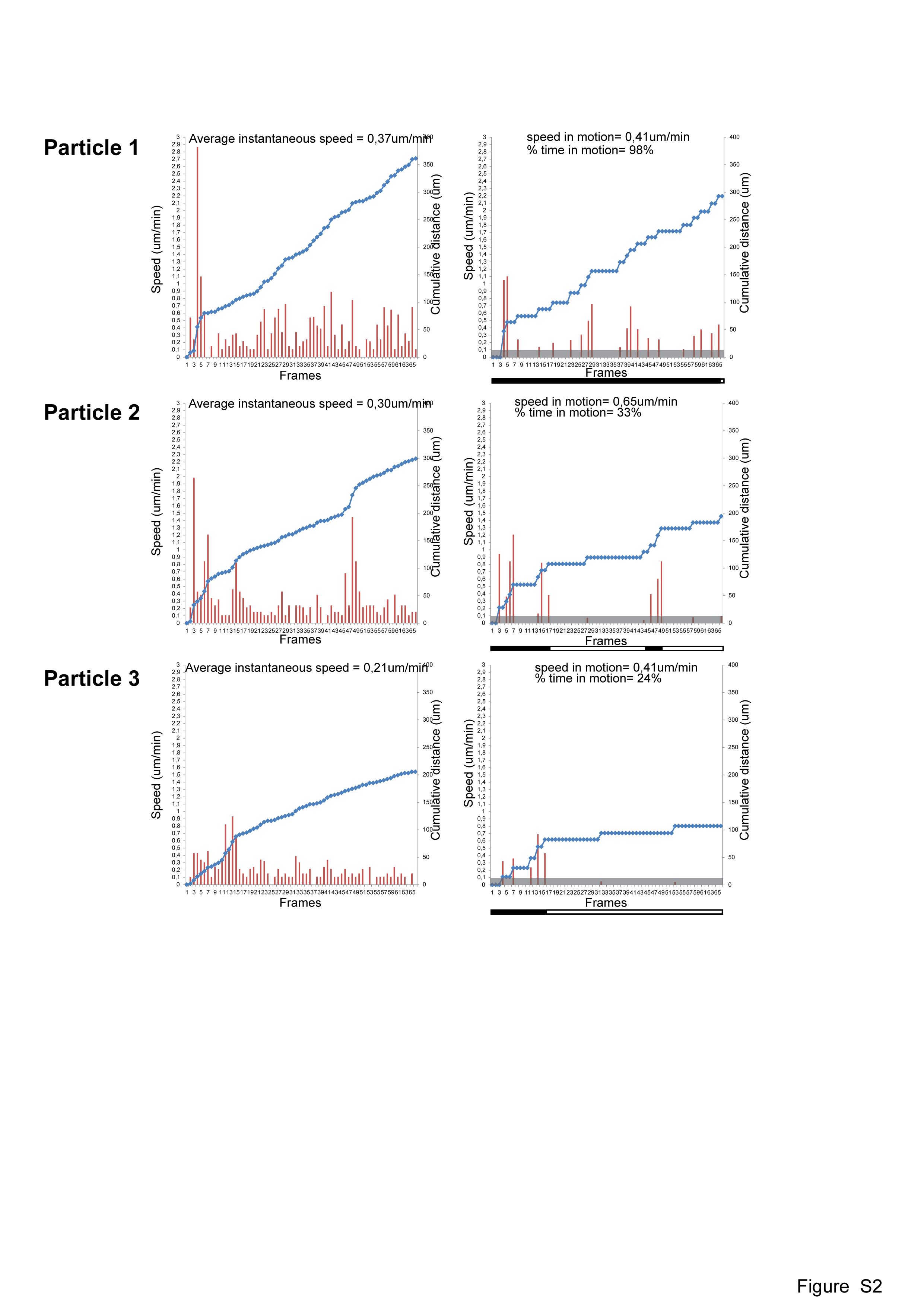

Supplement: Figure S2 — To compare particles behavior, the left panels represent the cumulative distance of raw position (blue line), the instantaneous speeds (red bars) and their average; the right panel shows the cumulative distance of positions corresponding to significative displacements (blue line), the speed for these displacements (red bars), the two types of periods (black and white bar and the values of speed and percentage of time in motion) obtained by Skypad analysis. (TIF) [file pone.0089073.s002.tif]

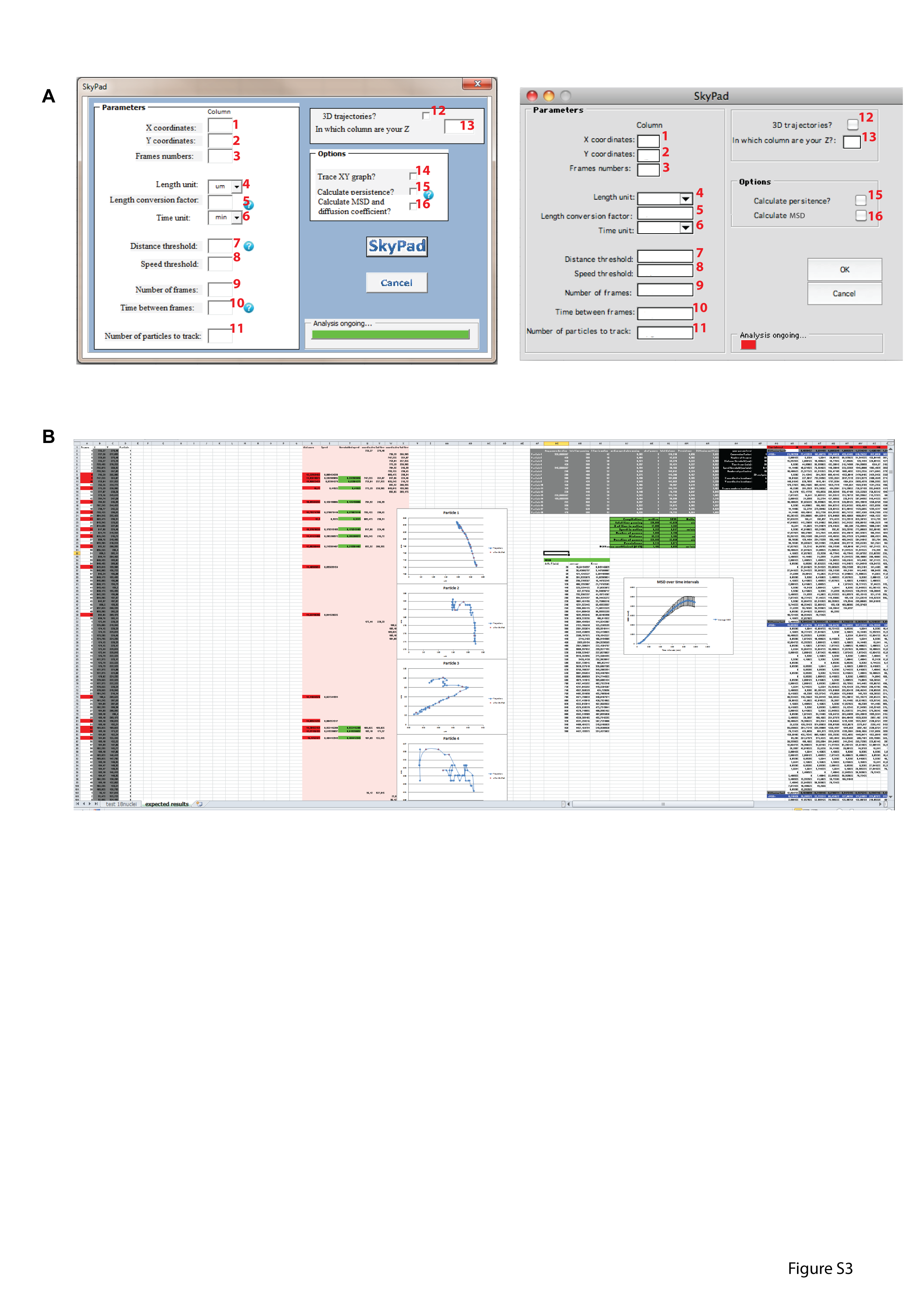

Supplement: Figure S3 — A: After pressing Ctrl+Shit+S or Cmd+Alt+S this window will appear in Windows (left panel) or MacOS (right panel). B: After analysis, the excel spreadsheet will be updated with the results. (TIF) [file pone.0089073.s003.tif]
